# Adding Insult to Injury: Asymptomatic Fat Embolism Identified on Computed Tomography

**DOI:** 10.5811/cpcem.2019.2.41888

**Published:** 2019-04-02

**Authors:** Malia J. Moore, Sophia Y. Liu

**Affiliations:** Carl R. Darnall Army Medical Center, Department of Emergency Medicine, Fort Hood, Texas

## Abstract

Fat embolism (FE) is a classically taught complication of long bone fractures, with the potential to cause high morbidity and mortality; however, it is rarely apparent on emergency department (ED) presentation or imaging. If recognized by the ED clinician, development of symptoms of FE may be avoided by early surgical fixation and potentially by corticosteroid administration.

## CASE PRESENTATION

**Image 1:**
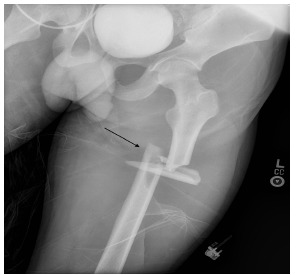
Radiograph of the left hip demonstrating segmental, displaced, and shortened left femur fracture (arrow).

**Image 2:**
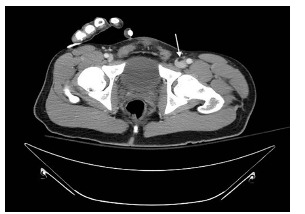
Computed tomography of the pelvis in axial view showing fat fluid level within the left common femoral vein (arrow).

**Image 3:**
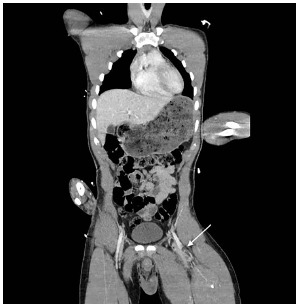
Computed tomography image of the chest, abdomen, and pelvis in coronal view showing fat fluid level within the left common femoral vein (arrow).

The patient was an adult male involved in a high-speed motor vehicle collision into a tree, with prolonged extrication due to vehicle deformity. On initial trauma exam, he had obvious left femur and left wrist deformities. A radiograph of the left hip revealed a femur fracture (Image 1). Computed tomography (CT) of the head, spine, chest, abdomen, and pelvis were performed. Imaging identified a fat embolism (FE) in the left common femoral vein (Images 2 and 3).

## DISCUSSION

CT images showed a fat fluid level within the left common femoral vein as a direct complication of femur fracture. FE is a rare syndrome in which fat globules migrate into vasculature, most commonly from traumatized adipose tissue or marrow-containing bone. FE can complicate a wide variety of conditions, most commonly long bone or pelvic fractures. Symptomatic fat emboli present with a triad of hypoxemia, neurologic abnormality, and petechial rash. Although care is largely supportive, the patient should be carefully monitored with pulse oximetry, frequent neurologic checks, and re-examination to promptly identify and treat development of FE syndrome. All symptoms are transient if not fatal.^1^,^2^

Early recognition of FE may prompt steps to avoid development of the clinical syndrome, such as early surgical fixation, which is preferred over traction, and corticosteroid administration. Corticosteroids have shown some benefit, especially in high-risk injuries with confirmed fat emboli; however, their routine empiric administration is controversial.^3^–^5^

**CPC-EM Capsule**

What do we already know about this clinical entity?

*Fat embolism (FE), a condition in which fat globules migrate into vasculature, is a known complication of long bone fractures that could lead to the development of FE syndrome*.

What is the major impact of the image(s)?

*The asymptomatic finding of FE in transit shown here is believed to be rare; however, its identification could help avoid the development of FE syndrome*.

How might this improve emergency medicine practice?

*Early identification of FE may prompt treatment and lessen the risk of morbidity and mortality associated with FE syndrome*.
